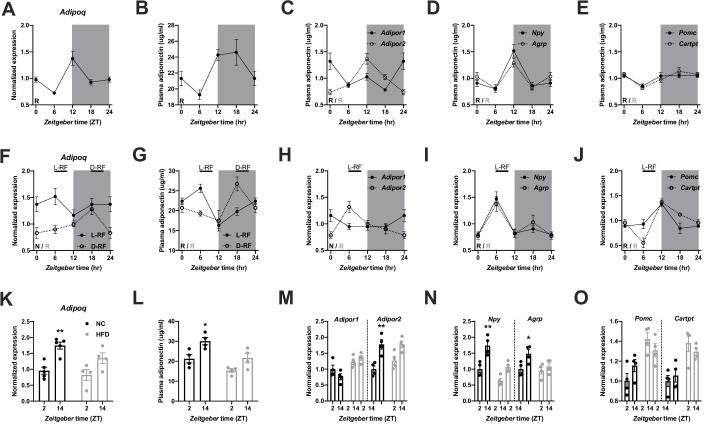# Correction: An adipokine feedback regulating diurnal food intake rhythms in mice

**DOI:** 10.7554/eLife.85366

**Published:** 2022-12-14

**Authors:** Anthony H Tsang, Christiane E Koch, Jana-Thabea Kiehn, Cosima X Schmidt, Henrik Oster

**Keywords:** Mouse

Tsang AH, Koch CE, Kiehn J-T, Schmidt CX, Oster H. 2020. An adipokine feedback regulating diurnal food intake rhythms in mice. *eLife* **9**:e55388. doi: 10.7554/eLife.55388.

Published 9 July 2020

In Figure 1 of this article, three sub-panels (C-E) have incorrect y-axis labels. Instead of “Plasma adiponectin (µg/ml)” it should read “Normalized expression” (same as in the corresponding panels H-J). This error likely occurred during type setting of the figure and harmonizing layout between the sub-panels. We have corrected this error and apologize for any confusion. Of note, this correction does not change the results or affect the scientific conclusions of the original report.

The article has been corrected accordingly.

Corrected figure:

**Figure 1:**
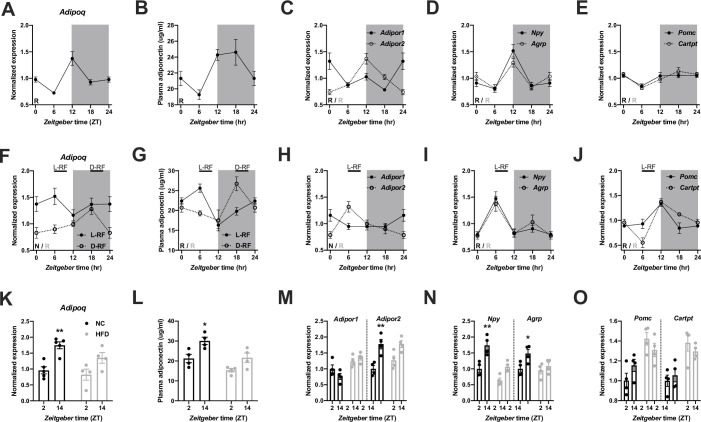


Original figure:

**Figure 2:**